# METABOLOMICS IN MEN WITH DISTINCT INFLAMMATION TRAJECTORIES FOLLOWING HIP FRACTURE REVEAL RESPONSE MECHANISMS

**DOI:** 10.1093/geroni/igz038.323

**Published:** 2019-11-08

**Authors:** Shabnam Salimi, Alice Ryan, Denise Orwig, Ann Gruber-Baldini, Jack Guralnik, Jay S Magaziner, March Hochberg

**Affiliations:** University of Maryland Baltimore School of Medicine, Baltimore, Maryland, United States

## Abstract

Introduction: Following hip fracture, men exhibit different trajectories of inflammation: high (HiInf) vs. low (LoInf). The purpose of this analyses was to compare metabolomics Between these groups. Methods: Seven men with HiInf and 7 with LoInf were randomly selected to quantify serum metabolites at baseline, 2, and 6 months following hip fracture in Baltimore Hip Study-7. We performed analysis of variance and mixed effects models. A p <0.05 was considered significant. Results: At baseline, men with HiInf had higher oxidative stress, lipid-related inflammatory markers, and antioxidant compounds compared to LoInf group. These markers were reduced in both groups at follow-up, consistent with reduced stress. However, the attenuation level was significantly different between the two groups. Kynurenate, that indicates activation of the immune system, was higher in HiInf group and remained elevated over 6 months follow-up. Polyamines with antioxidant activity and boosting autophagy activities were declined more significantly in the LoInf group presumably indicating more response to bodily anti-oxidant activity compared to the HiInf group. Arachidonic acid-derived eicosanoids, mediators of the immune response, were significantly elevated at baseline in the LoInf while oxidative stress markers were increased more in HiInf group. Branched-chain amino acids (BCAAs), the essential amino acids abundant in muscle, were elevated at baseline in the LoInf compared to HiInf group. Conclusion: Both groups showed an increase in anti-oxidative stress while LoInf group showed more reduction in oxidative stress and immune cell activity. Reduced intermediate compounds of BCCA suggest that their catabolism was attenuated following hip fracture in LoInf.

